# Major depression disorder and heart failure: A two-sample bidirectional Mendelian randomization study

**DOI:** 10.1371/journal.pone.0304379

**Published:** 2024-05-29

**Authors:** Wei Liu, Quan Lin, Zongjing Fan, Jie Cui, Yang Wu

**Affiliations:** Department of Cardiology, Dongfang Hospital, Beijing University of Chinese Medicine, Beijing, China; Fundación Universitaria del Área Andina, COLOMBIA

## Abstract

**Objective:**

To determine whether a bidirectional causal relationship exists between major depressive disorder (MDD) and heart failure (HF).

**Methods:**

Our two-sample bidirectional Mendelian randomization (MR) study consisted of two parts. In the first part, we conducted a forward MR analysis where MDD was considered as the exposure and HF as the outcome. In the second part, a reverse MR analysis was performed, treating HF as the exposure and MDD as the outcome. Summary data on MDD and HF were obtained from the IEU Open GWAS database.

**Results:**

Based on the results of the MR-Egger regression intercept test, there was no evidence of horizontal pleiotropy in this study. Furthermore, the IVW results consistently suggested estimates of causal effect values. The findings revealed that individuals with MDD had a 16.9% increased risk of HF compared to those without MDD (OR = 1.169, 95%CI: 1.044–1.308, *P* = 0.007). However, there was no evidence to support that HF would increase the risk of MDD (OR = 1.012, 95%CI: 0.932–1.099, *P* = 0.773). Heterogeneity in SNPs of MDD and HF was observed through the heterogeneity test and funnel plot. Additionally, the leave-one-out method did not identify any instances where a single SNP was biased toward or dependent on causation.

**Conclusion:**

Our study provides evidence supporting a one-way causal relationship between MDD and HF. Specifically, MDD increases the risk of developing HF. However, our findings did not provide any evidence suggesting that HF increases the risk of developing MDD.

## 1 Introduction

Major depressive disorder (MDD), sometimes referred to simply as depression, is a heterogeneous mental disorder characterized by prolonged low mood, pessimism, and anhedonia [1]. MDD is considered one of the most pressing mental health problems because the global incidence has increased by about 50% in the last 30 years, affecting more than 264 million people [2]. According to the World Health Organization, depression is a major contributor to the global burden of disease and a leading cause of mental and physical disability [3].

Heart failure (HF) occurs secondary to a variety of cardiovascular diseases and is the final stage of cardiac impairment. Symptoms such as dyspnea, exertion, poor exercise tolerance, and fluid retention occur, which seriously affect the patient’s quality of life. HF poses a serious burden on global public health. It is estimated that there are 37.7 million people affected by HF, resulting in 4.2 million disabilities [4]. Importantly, depression increases the risk of HF in healthy people. A meta-analysis found that people with depression had a 46% increased risk of cardiovascular disease compared to healthy people [5]. On the other hand, HF is associated with a higher risk of depression. Another meta-analysis yielded a finding that the prevalence of depression in HF patients was 29% [6]. The aforementioned studies delved into the discussion of risk factors and their findings indicated a correlation between MDD and HF, without establishing a causation. However, they did not confirm a bidirectional causal relationship between MDD and HF.

Mendelian randomization (MR) research is a process that utilizes single nucleotide polymorphisms (SNPs) as instrumental variables (IV) to establish models and infer and evaluate causal effects [7]. The random assignment of exposed IVs during conception eliminates unobserved confounders, allowing MR studies to assess causal relationships between exposure and disease [8]. In recent years, genome-wide association studies (GWAS) have rapidly accumulated millions of published data on associations between genetic variants and phenotypes. By leveraging this data, the impact of risk factors on outcomes can be assessed without the need for additional studies to recruit new patients. Currently, MR studies are widely employed for causal inference in various diseases. The objective of this study was to investigate the bidirectional causal relationship between MDD and HF using MR. The aim is to provide valuable insights for the prevention and treatment of MDD and HF.

## 2 Methods

### 2.1 Study design

**Fig 1:**
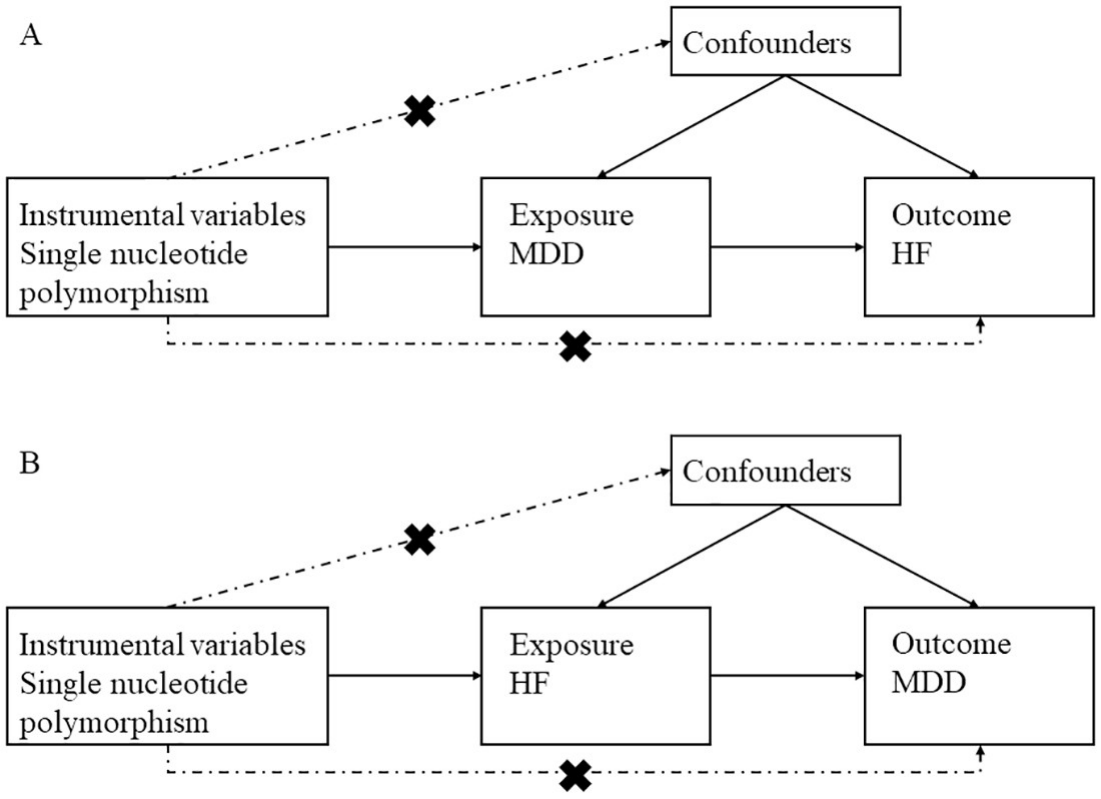
Description of the study design in this bidirectional MR study. (A) The forward MR analyses, with MDD as exposure and HF as the outcome. (B) The reverse MR analyses, with HF as exposure and MDD as the outcome. MDD, major depression disorder; HF, heart failure; MR, Mendelian randomization.

To investigate the bidirectional relationship between MDD and HF, we conducted two MR analyses, which were divided into forward and reverse analyses [7]. In the forward analysis, we examined the causal effect of MDD on HF, while in the reverse analysis, we explored the causal effect of HF on MDD. These analyses were performed utilizing GWAS data. The two-sample MR method was employed to assess the causal effects (Fig 1). As our study solely utilized publicly available summary data, ethical review was not required.

### 2.2 Testing MR assumptions

MR studies rely on the fulfillment of three core hypotheses [8]: (1) A strong relationship exists between genetic variation and the exposure of interest; (2) Genetic variation is not associated with potential confounding factors; (3) Genetic variation, apart from its influence on the exposure, is not associated with the outcome of interest (Fig 1). We assessed assumption one using the F-statistic, calculated as F = (R^2^ × [n– 1 − k])/ ([1 −R^2^] × k), where R^2^ represents the proportion of exposure variance explained by genetic variation, n is the sample size, and k is the number of SNPs [9, 10]. Typically, an F > 10 is considered satisfactory for assumption one, indicating no weak instrumental variable bias. Testing hypotheses 2 and 3 is complicated due to the presence of unknown potential confounding variables. As a result, we estimated the MR Egger’s regression coefficients and examined if the intercept was significantly different from zero, which would suggest the presence of horizontal pleiotropy [11, 12].

### 2.3 Data sources

The genetic variants associated with MDD were obtained from a study published in Nature Neuroscience in 2019 [13]. The study included a total of 170,756 individuals with MDD as cases and 329,443 controls of European ancestry. The dataset comprised 11,734,353 SNPs. The genetic variants associated with HF were sourced from a study published in Nature Communications in 2020 [14]. The HF study involved 47,309 cases and 930,014 controls of European ancestry, with a total of 7,773,021 SNPs analyzed. The MDD and HF analysis data were aggregated and are available in the IEU Open GWAS database (https://gwas.mrcieu.ac.uk/).

### 2.4 Screening SNP

The selection of genetic instruments followed specific criteria: (1) Significant association with the exposure at a *P*-value<5×10^−8^, (2) A linkage disequilibrium r^2^<0.001, or a genetic distance≤10000kb, (3) Minimum allele frequency>0.01, (4) Exclusion of SNPs with palindromic intermediate allele frequencies, and (5) Independence of the SNPs from each other. After applying these screening and extraction criteria, the study identified a total of 50 SNPs associated with MDD and 12 SNPs associated with HF. The β values of the effect alleles ranged from -0.0620 to 0.0704 for MDD, with the highest absolute value of |β| being 0.0306 for HF. Regarding the effect alleles for HF, the β values ranged from -0.0666 to 0.1620, with the highest absolute value of |β| being 0.0151 for MDD. The F-statistic for all SNPs in the study exceeded 10, indicating the absence of weak instrumental variable bias.

### 2. 5 Statistical analysis

We utilized the Wald ratio to estimate the causal impact of each IV. We employed an inverse-variance weighted (IVW)-based multiplicative random-effects model, and Cochran’s Q value did not yield a significant result (*P* > 0.05) [15]. The primary analysis employed the IVW technique to assess the association between genetically predicted MDD-associated features and the risk of AF [16]. The combined results from single and multiple SNP analyses were visualized using forest and scatter plots. In the forest-like figure, the effect estimates from the single SNP and multiple SNP analyses are presented side by side. Additionally, in the scatterplot, estimated regression lines from the multiple SNP analysis are overlaid to compare the impact of single SNPs on exposure and outcomes.

Next, we conducted a series of sensitivity analyses. In the first analysis, we employed the weighted median method to assess relevance [17]. Additionally, we utilized multiple methods including MR-Egger, simple mode, and weighted mode to provide estimates under a wider range of conditions. Moreover, we evaluated horizontal multiplicity through MR-Egger intercept data [12]. The challenges posed by complex behavioral exposures make it difficult for commonly used pleiotropy robust methods to yield estimates with the correct direction of effect, highlighting the struggle in detecting systematic pleiotropy. These estimators often assume that each SNP has a unique pleiotropic effect. Our study exercised caution in interpreting results within such settings [18]. To further investigate the robustness of our findings, we conducted a leave-one-out analysis using the IVW MR method. Finally, in order to assess heterogeneity, we examined funnel plots.

All of the analyses were performed using R (version 4.3.1) and R Studio (version 2022.06.1+524). Statistical significance was defined as *P* < 0.05.

## 3 Results

### 3.1 The casual effect of MDD on HF

**Fig 2:**
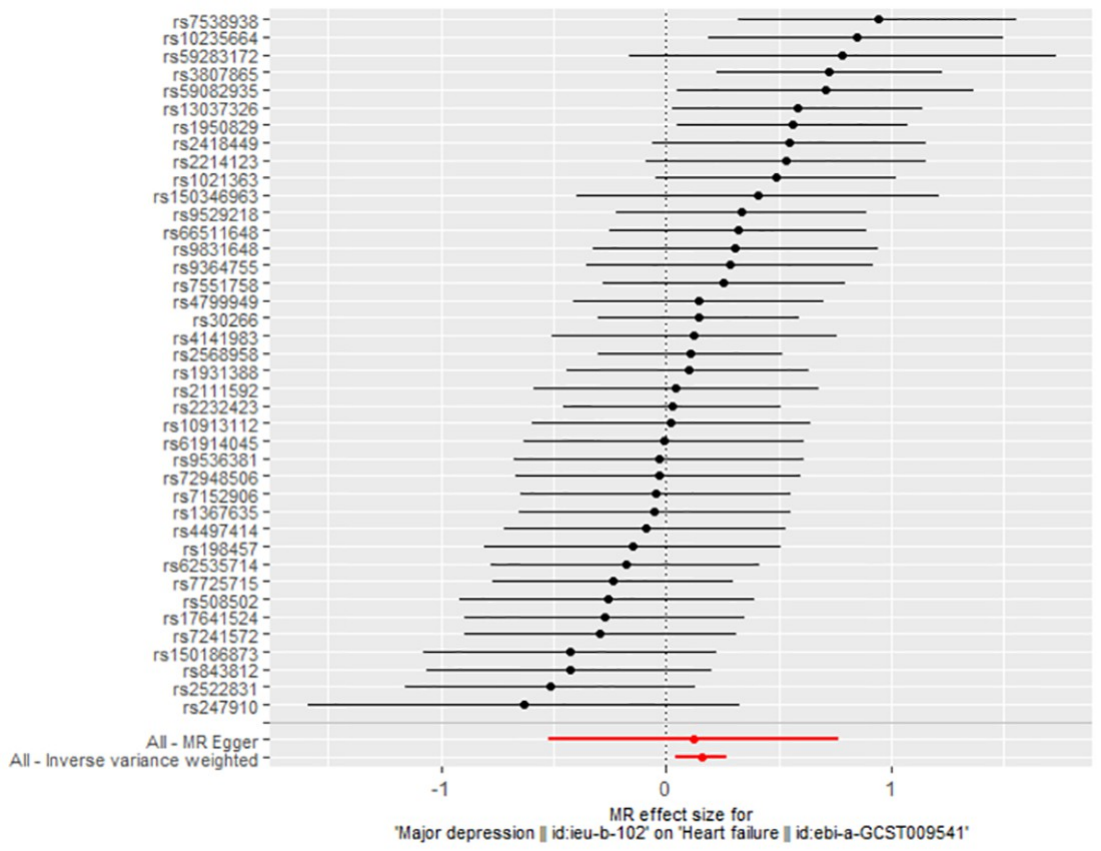
The forest plot of MR analyses for MDD on HF.

**Fig 3:**
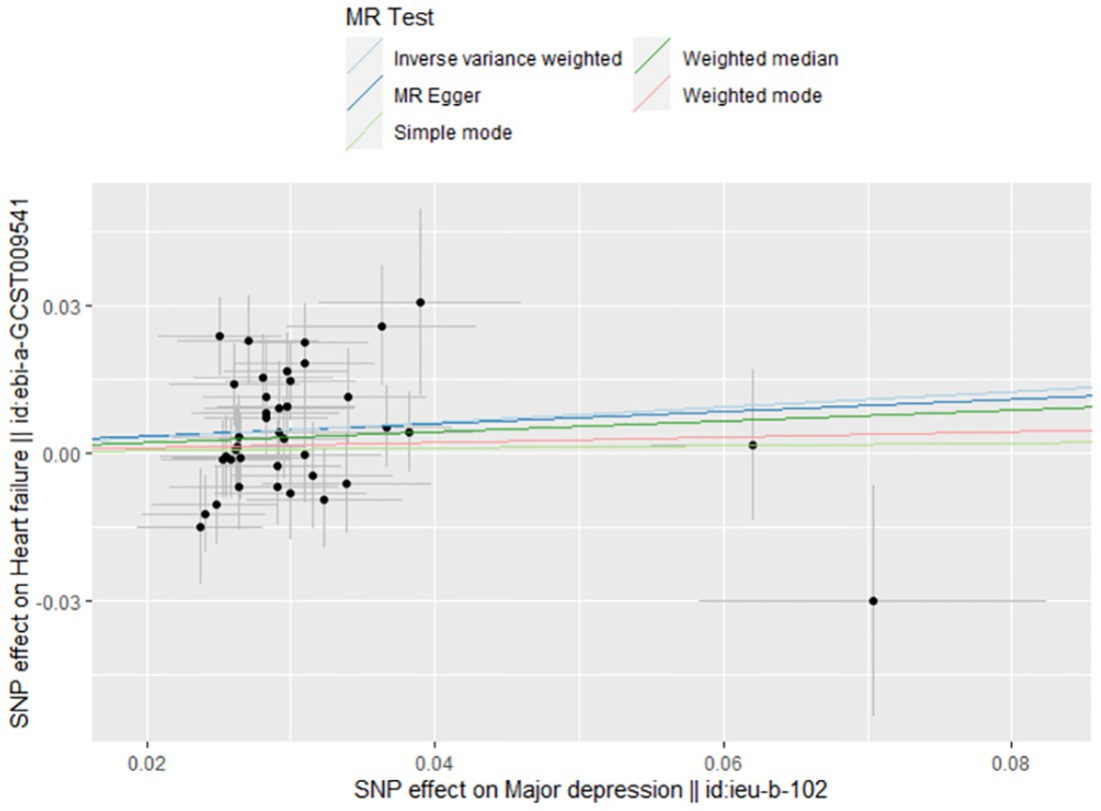
The scatter plot of MR analyses for MDD on HF.

**Fig 4:**
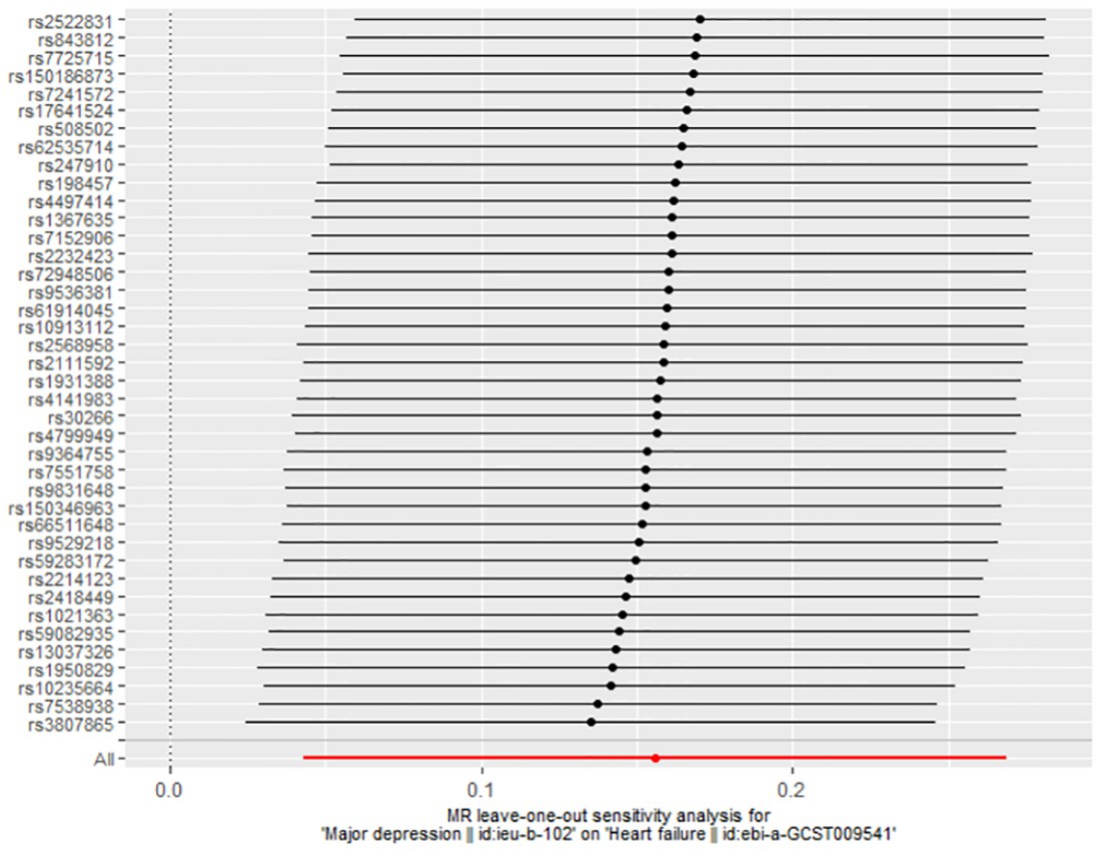
MR leave-one-out analyses for MDD on HF.

**Fig 5:**
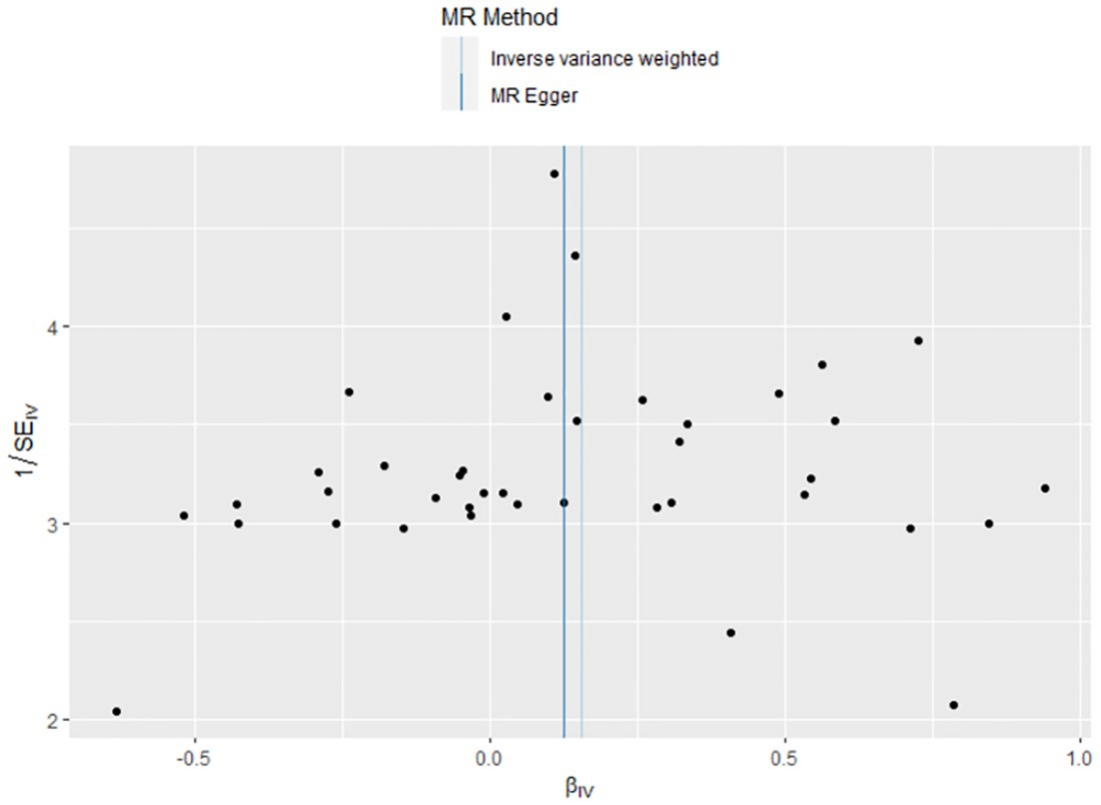
The funnel plot of MR analyses for MDD on HF.

The association between genetically predicted MDD-related features and the risk of HF was assessed using the random-effects IVW technique. In our conventional MR analysis using the IVW method, we found evidence suggesting a potential causal relationship between MDD and HF (odds ratio [OR]: 1.169, 95% CI 1.044–1.308; *P* = 0.007) (Table 1). The forest plot (Fig 2) and scatter plot (Fig 3) both visually represented the causal estimates obtained from individual IVs. Additionally, we performed an MR-Egger regression test to detect pleiotropy and found no significant indication of it (intercept = 0.001, *P* = 0.922). We further observed that even after removing other SNPs, our results remained robust, and no SNP showed a significant impact on the estimation of causal effects (Fig 4). The heterogeneity test results for both IVW and MR-Egger, along with the funnel plot (Fig 5), indicated significant heterogeneity among the SNPs.

**Table 1:** Influence of MDD traits on HF by MR models.

Method	Number of SNPs	OR	95%CI	*P*-Value
Inverse variance weighted	40	1.169	1.044–1.308	0.007
Weighted median	40	1.115	0.967–1.286	0.134
MR Egger	40	1.132	0.594–2.157	0.708
Simple mode	40	1.025	0.745–1.411	0.881
Weighted mode	40	1.057	0.795–1.405	0.707

### 3.2 The casual effect of HF on MDD

**Fig 6:**
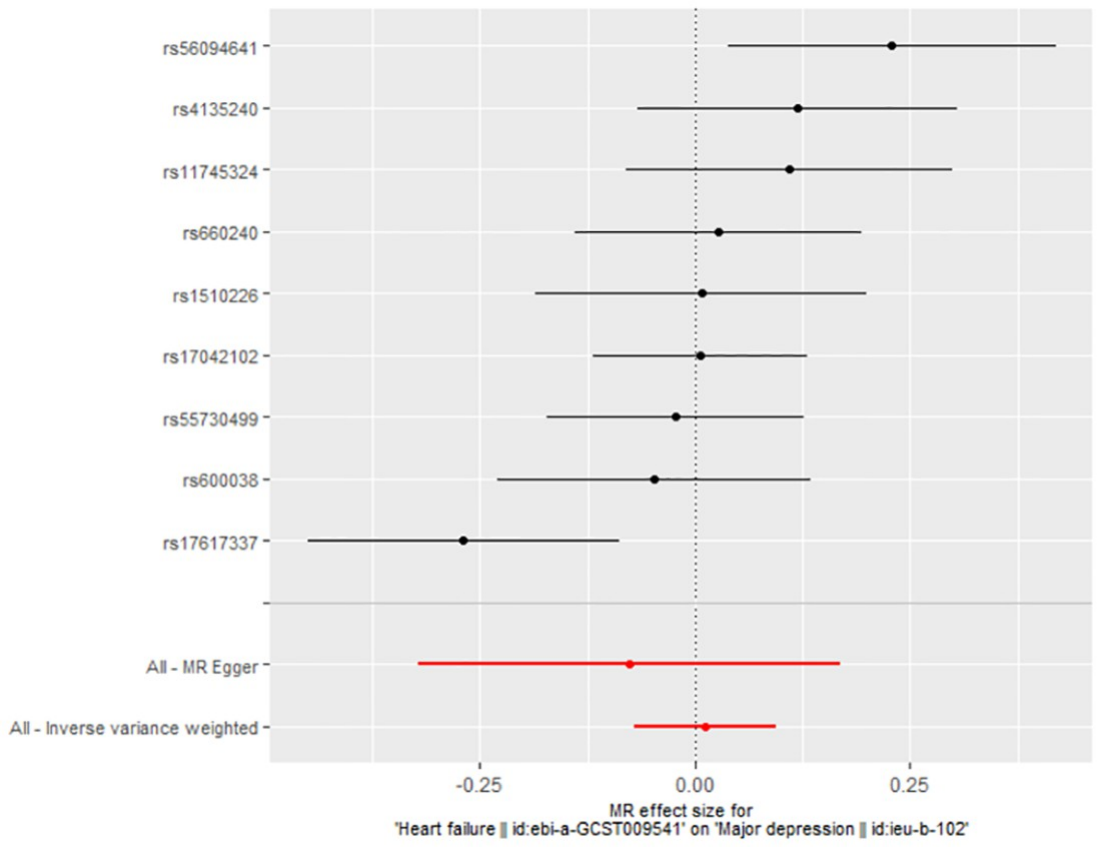
The forest plot of MR analyses for HF on MDD.

**Fig 7:**
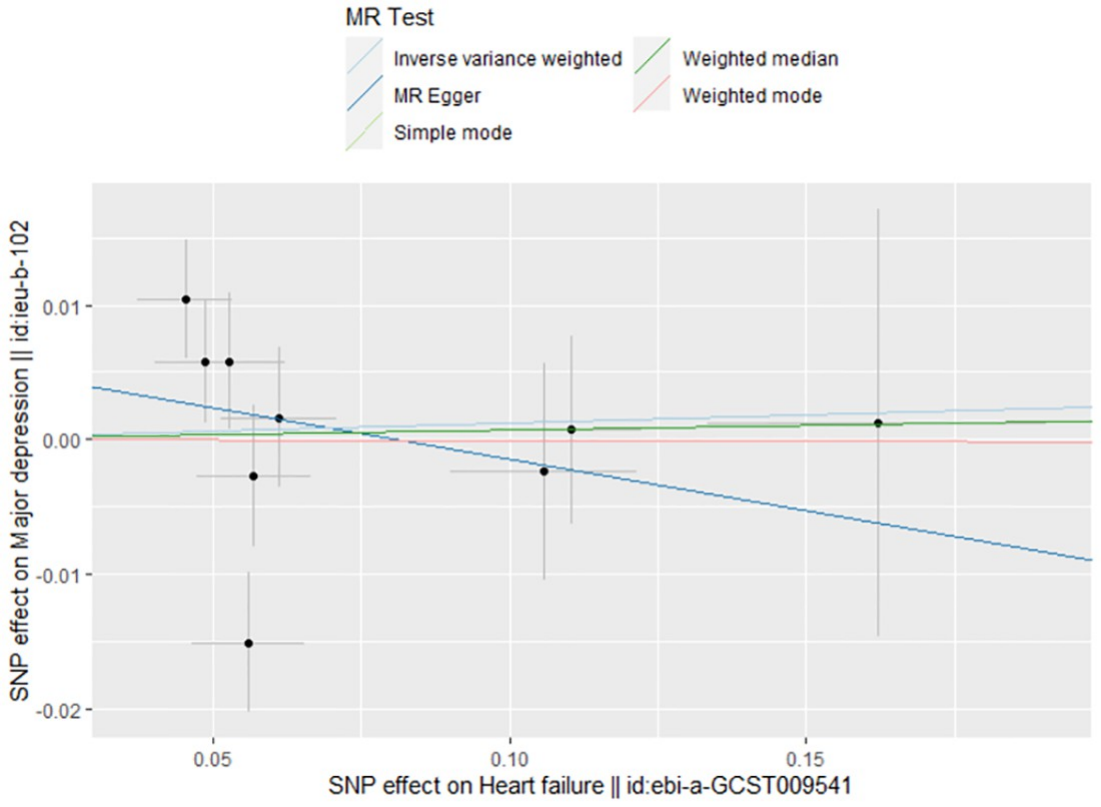
The scatter plot of MR analyses for HF on MDD.

**Fig 8:**
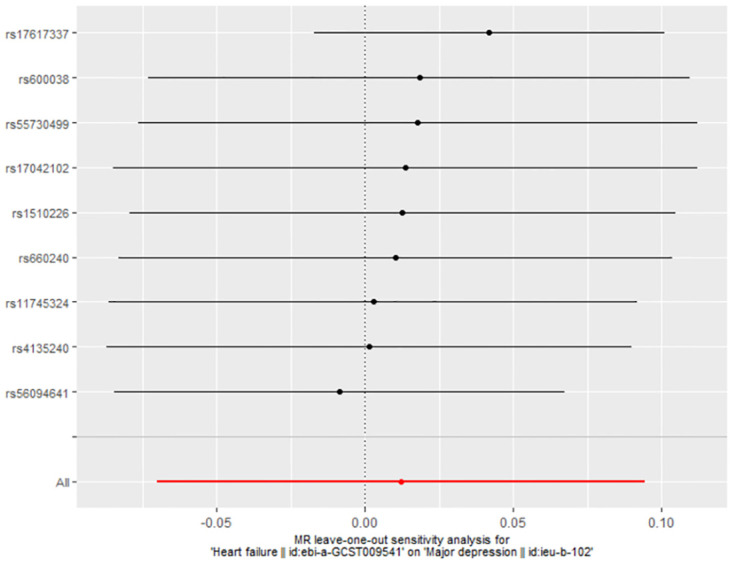
MR leave-one-out analyses for HF on MDD.

**Fig 9:**
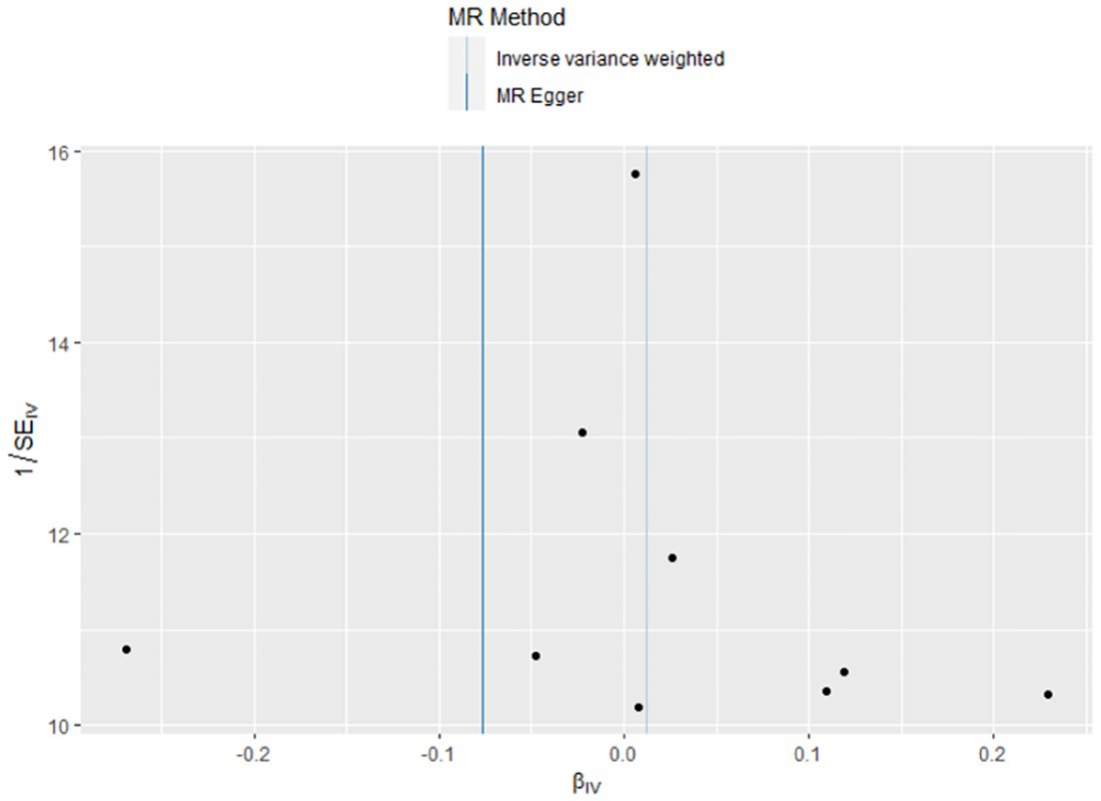
The funnel plot of MR analyses for HF on MDD.

The link between genetically predicted MDD-related features and HF risk was assessed using the random-effects IVW technique. However, in the conventional MR analysis by the IVW method, we did not find evidence for a potential causal relationship between HF and MDD (OR: 1.012, 95% CI 0.932–1.099; *P* = 0.773) (Table 2). Both the forest plot (Fig 6) and scatter plot (Fig 7) presented the causal estimates derived from individual IVs. Importantly, we observed no pleiotropy, as indicated by the MR-Egger regression test with an intercept of 0.006 (*P* = 0.477). Furthermore, we conducted additional analyses by removing other SNPs, confirming the robustness of the results, as no SNP significantly influenced the estimation of causal effects (Fig 8). The heterogeneity tests of IVW and MR-Egger, along with the funnel plot (Fig 9), provided further support for the significant heterogeneity among the SNPs.

**Table 2:** Influence of HF traits on MDD by MR models.

Method	Number of SNPs	OR	95%CI	*P*-Value
Inverse variance weighted	9	1.012	0.932–1.099	0.773
Weighted median	9	1.007	0.931–1.088	0.863
MR Egger	9	0.927	0.725–1.185	0.562
Simple mode	9	0.999	0.897–1.112	0.987
Weighted mode	9	0.999	0.907–1.100	0.985

## 4 Discussion

The study utilized bidirectional MR to examine the genetic association between MDD and HF. Our analysis conclusively demonstrated a potential one-way causal association, where MDD was identified as a risk factor for developing HF, while HF did not exhibit a statistically significant effect on MDD.

The results of the present study indicated that individuals with MDD had a 16.9% increased risk of developing HF (OR = 1.169, 95% CI: 1.044–1.308), which agrees with several epidemiological findings. It is well-established that depression, as a conventional risk factor, can elevate the risk of major morbidity and mortality by 2 to 3 times in patients with HF [19]. A meta-analysis highlighted the significant association between depression and the development of congestive heart failure [20]. Moreover, a specific study demonstrated that patients with HF who had comorbid MDD exhibited a higher rate of readmissions when compared to those without depression [21]. This finding underscores the significance of depression as an independent risk factor for multiple all-cause readmissions in individuals hospitalized with congestive HF. However, there are exceptions. One study found that while MDD may increase the risk of most types of cardiovascular disease, including arrhythmia, stroke, and high blood pressure, it does not appear to affect the risk of HF [22]. Furthermore, multiple studies have identified HF as a risk factor for MDD. In a meta-analysis examining depression in individuals with HF, it was revealed that 11% to 25% of outpatients and 35% to 70% of inpatients experienced depression. Additionally, this investigation, along with colleagues, discovered a progressive increase in depressive symptoms corresponding to the severity of the HF diagnosis. Specifically, the prevalence of depressive symptoms ranged from 11% in patients categorized as New York Heart Association (NYHA) functional class I to 42% in patients classified as NYHA class IV [23]. However, our study did not find a causal relationship between HF and MDD. We found no direct evidence suggesting that HF increases the odds of developing MDD. Similarly, one study found that the risk of developing mental health disorders, such as bipolar disorder and schizophrenia, was associated with HF, rather than MDD [24]. Therefore, we hypothesize that the presence of confounding factors may have influenced the results of the epidemiological study. And, it cannot be ruled out that certain HF subtypes may have a potential impact on MDD. The possibility that specific types of HF may be risk factors for MDD is a direction for future research.

The mechanism by which MDD leads to HF has yet to be fully understood. Despite extensive research efforts, significant progress has been made in elucidating this relationship. The shared pathophysiological feature of inflammation appears to be a common characteristic linking depression and HF [25]. Notably, MDD is associated with elevated levels of inflammatory biomarkers. A meta-analysis comprising individuals both with and without cardiovascular disease demonstrated a correlation between elevated levels of C-reactive protein (CRP), IL-1, and IL-6, and depression [26]. Additionally, other studies have reported an association between elevated levels of tumor necrosis factor-alpha (TNF-α) and monocyte chemotactic protein-1 (MCP-1) and depression [27]. Similarly, an analysis of data from the Cardiovascular Health Study revealed a significant association between depression and elevated levels of inflammatory and fibrosis markers [28]. Inflammation has been implicated in the pathogenesis of various types of HF and may contribute to ventricular remodeling, resulting in increased fibrosis and deterioration of cardiac function [29, 30]. In patients with HF, both short- and long-term cardiovascular mortality have been linked to the presence of interleukin-6 [31]. As a result, depression may lead to elevated levels of inflammatory markers in the body, thereby increasing the risk of developing HF. Additionally, depression can also contribute to oxidative stress [32, 33] and dysfunction in the autonomic nervous system [34, 35], further escalating the risk of HF. To comprehensively understand the molecular processes underlying these mechanisms, further research is necessary. A deeper understanding of these processes will facilitate the discovery of new preventive and therapeutic measures.

This study has several strengths. Firstly, the bidirectional MR analysis provided evidence for a unidirectional causal relationship between MDD and HF. Secondly, the utilization of MR analysis, which considers confounders and reverse causality, ensures accurate estimation of causal effects. However, it is important to acknowledge the limitations of this study. The GWAS data used in this study represents individuals of European descent. Therefore, it is crucial to validate these findings in other ethnic groups to ensure their generalizability beyond European populations. Furthermore, it is worth noting that our discussion regarding the relationship between MDD and HF is limited to a genetic causality perspective. The interplay between environmental and genetic factors plays a significant role in disease development. Additional studies utilizing various analytical designs to account for potential confounding factors are necessary to confirm and further explore our findings.

## 5 Conclusion

In this study, we sought to establish a clear causal link between MDD and HF, demonstrating that MDD potentially increases the risk of HF. However, the evidence supporting the reverse relationship, where HF causes MDD, is limited. Consequently, it is crucial to acknowledge the impact of depression in the prevention and treatment of HF. Attention to MDD within the population should be heightened, and interventions related to MDD should be integrated into HF prevention strategies. Moreover, it is imperative to prioritize cardiovascular disease prevention in individuals diagnosed with MDD.
